# Studying Social Cognition in Patients with Schizophrenia and Patients with Frontotemporal Dementia: Theory of Mind and the Perception of Sarcasm

**DOI:** 10.1155/2008/157356

**Published:** 2008-04-11

**Authors:** Mary H. Kosmidis, Eleni Aretouli, Vassilis P. Bozikas, Maria Giannakou, Panayiotis Ioannidis

**Affiliations:** ^1^School of PsychologyAristotle University of ThessalonikiThessalonikiGreece; ^2^2nd Psychiatry DepartmentAristotle University of ThessalonikiThessalonikiGreece; ^3^2nd Neurology DepartmentAristotle University of ThessalonikiThessalonikiGreece

**Keywords:** Social cognition, theory of mind, schizophrenia, frontotemporal dementia

## Abstract

We investigated social cognition and theory of mind in patients with schizophrenia and in patients with frontotemporal dementia in order to elucidate the cognitive mechanisms involved in the breakdown of these skills in psychiatric and neurological patients. Our tasks included videotaped scenarios of social interactions depicting sincere, sarcastic and paradoxical remarks, as well as lies. We found impaired performance of the schizophrenia group on all theory of mind conditions despite their intact understanding of sincere statements. In contrast, the FTD group performed poorly only when they had to rely on paralinguistic cues indicating sarcasm or lies, and not on paradoxical remarks or sarcasm when given additional verbal cues. Our findings suggest that, while current deficits in social and interpersonal functioning in patients with FTD may reflect a decrement in previously acquired skills, similar deficits in patients with schizophrenia may reflect an altogether inadequately learned process.

## 1. Introduction

Social cognition is an umbrella term that comprises a variety of skills which make successful social interaction possible. One of these skills, theory of mind (ToM), is the ability to perceive the intentions and beliefs of others. This skill has received considerable research attention recently, particularly with respect to schizophrenia [1,5,10,11], as it may help to explain these patients' poor social and interpersonal functioning. In fact, some theorists have suggested that positive symptoms are the result of erroneous attribution of mental representations in schizophrenia [10,11] or the inability to inhibit personal beliefs and subsequent attribution of one's own beliefs to others. Other researchers have suggested that patients with schizophrenia do not lack ToM but tend to overuse or misuse it [1], interpreting others' intentions beyond what the data support [5]. Regardless of symptom subtype (positive or negative), patients with schizophrenia appear to have a poor understanding of the subtle cues typically used in a conversation [7].

Individuals with frontotemporal dementia (FTD) also demonstrate unusual and inappropriate social behaviour in the early stages of the disorder, even before the diagnosis is confirmed. They exhibit deficits in empathy and inhibition of behavior, impulsivity, stereotypical behaviors and changes in appetite [4] with little cognitive decline [13]. In fact, their behavioral changes often lead to an erroneous diagnosis of schizophrenia or bipolar disorder, rather than dementia [6,12]. Sever-

ISSN 0953-4180/08/$17.00 © 2008 – IOS Press and the authors. All rights reserved al studies have reported deficits in the perception and processing of emotions in patients with FTD [4,19,20], implicating the ventromedial frontal lobes [14], an area thought to be involved in social functioning [3,9]. Interestingly, ToM and social cognition were found to be independent of overall neuropsychological status [15]. Therefore, changes in social behavior may reflect difficulties in the perception of emotional cues in FTD.

We sought to investigate the cognitive mechanisms involved in one aspect of social cognition, namely, ToM. More specifically, we were interested in comparing two patient groups with known frontal and temporal dysfunction on tasks tapping into ToM, particularly the ability to infer the meaning of a conversational interaction in which the speaker's true opinions are not literal, as often occurs in daily discourse.

## 2. Method

### 2.1. Participants

Our sample comprised 28 patients diagnosed with schizophrenia (according to DSM-IV criteria [2] and confirmed with the Greek version (translation-adaptation to the Greek language by S. Beratis) of the Mini International Neuropsychiatric Interview (4.4) (MINI) [20]) and their age- and education-matched healthy control group ($n = 26$). We also examined nine FTD patients [all fulfilled the Lund-Manchester consensus criteria for frontal variant frontotemporal dementia [18] and were at an early stage of the disorder] and their age- and education-matched healthy control group ($n = 10$). Table 1 lists demographic characteristics of the groups. Exclusion criteria for the patient groups included other psychiatric or neurological diagnoses, a diagnosis of a developmental disorder, a history of head injury with loss of consciousness, alcohol or drug abuse during the six-month period prior to testing, non-native speakers of Greek, and any physical illness that may have affected cognitive performance. The same exclusion criteria, plus a history of any psychiatric or neurological diagnosis, applied to the healthy participants.

### 2.2. Procedure

In order to test the ability to perceive sarcasm, we used two subtests of a broader test of ToM, the Perception of Social Inference Test (PESIT), which we developed for the Greek population (based on the Awareness of Social Inference Test [16,17]). The items on these subtests were videotaped scenarios played by two actors. In the Social Inference-Minimal (SI-M) subtest, we asked participants to view a series of brief scenarios of a social interaction in which one of the actors made a sincere, sarcastic or paradoxical conversational remark. Comprehension was assessed by three questions following each dialogue, assessing the speaker's emotional state, the meaning of his/her remarks, his/her beliefs and intentions and the message he/she thought that the other person got from his/her remarks. Table 2a lists examples of a sarcastic and a paradoxical remark.

The Social Inference–Enriched (SI-E) subtest differed from the previous in that contextual cues (in the form of a prologue or dialogue, which revealed the speaker's true thoughts) were provided in addition to the paralinguistic features (namely, prosody, facial expressions and any subtle upper body movement that came naturally as the speaker made his/her comment). Participants were required to assess the speaker's emotional state, the meaning of his/her conversational remarks, his/her beliefs and intentions, and the message he/she thought that the other person got from his/her remarks. Through this subtest, we examined the ability to use contextual knowledge to understand a scenario, in which a false remark is made either sarcastically (meant to imply the opposite meaning, thus amplifying the inferred truth) or as a lie (meant to conceal the truth). Table 2b lists examples of items on this subtest showing a sarcastic remark and a lie.

The aforementioned subtests were administered in the same order for all participants (first SI-M, then SI-E). Within each subtest, the items were also presented in the same order to all participants, regardless of their specific nature (SI-M: first sincere items, then sarcastic, and finally paradoxical; SI-E: first sarcastic, then lies).

## 3. Results

### 3.1. Schizophrenia

Patients with schizophrenia performed more poorly than their healthy control group on both conditions of sarcasm (minimal: $U = 70.5$, $p < 0.001$; enriched: $U = 156.0$, $p < 0.001$), on the paradoxical statements ($U = 36.5$, $p < 0.001$) and on lies ($U = 69.5$, $p < 0.001$), despite their unimpaired performance on the sincere statements condition ($U = 259.0$, $p > 0.05$). Table 1 lists means and standard deviations for the performance of all groups on all tests.

Table 1
Means (*SDs*) of demographic characteristics and test performance by group

| Variable | Group | | | | | | | |
|---|---|---|---|---|---|---|---|---|
| | Schizophrenia ($n = 28$) | | Healthy controls ($n = 26$) | | Frontotemporal dementia ($n = 9$) | | Healthy controls ($n = 10$) | |
| | Median | Mean (*SD*) | Median | Mean (*SD*) | Median | Mean (*SD*) | Median | Mean (*SD*) |
| Age (years) | – | 36.3 (7.5) | – | 33.8 (9.8) | – | 64.6 (7.8) | – | 64.0 (7.2) |
| Education (years) | – | 11.9 (2.3) | – | 12.9 (2.5) | – | 7.8 (4.5) | – | 9.6 (2.8) |
| Duration of illness (years) | – | 11.4 (7.8) | – | – | – | 2.7 (1.6) | – | – |
| Social Inference-Minimal | | | | | | | | |
| Sincere | 16.0 | 14.0 (4.1) | 16.0 | 16.0 (2.2) | 14.0 | 12.9 (4.6) | 16.5 | 15.6 (2.8) |
| Sarcastic | 12.0 | 10.2 (5.7)** | 18.0 | 17.3 (1.3) | 4.0 | 4.7 (4.3)** | 13.0 | 12.3 (4.5) |
| Paradoxical | 12.0 | 11.4 (5.6)** | 24.0 | 22.1 (3.7) | 14.0 | 13.5 (7.0) | 19.0 | 17.7 (5.9) |
| Social Inference-Enriched | | | | | | | | |
| Sarcastic | 9.5 | 9.0 (3.7)** | 14.0 | 14.1 (1.6) | 10.0 | 7.9 (4.2) | 13.5 | 12.9 (2.2) |
| Lies | 10.0 | 9.6 (4.8)** | 15.0 | 13.9 (7.0) | 7.0 | 7.9 (6.4)* | 11.0 | 10.8 (3.8) |

*$p < 0.005$.
*$p < 0.001$.

Table 2
Examples of items from (a) the Social Inference-Minimal (SI-M) and (b) the Social Inference-Enriched subtests

| (a) Social Inference-Minimal subtest | |
|---|---|
| Sarcastic remark | Paradoxical remark |
| *Nick*: "Did you have a good time yesterday?"<br>*Mary*: "What can I say, it was *fantastic*. . . " | *Mary*: "Do you like my new haircut?"<br>*Nick*: "Did you *pay* for that?" |
| **(b) Social Inference-Enriched subtest** | |
| Sarcastic remark | Lie |
| *Mary* (speaking on the phone): Nick and I were looking at the photographs yesterday and laughing. Even *he* thought we looked awful.<br>*Nick* (enters): Hi, Mary. How's it going?<br>*Mary*: I was just saying how *great* your photographs were. | *Mary* (speaking on the phone): "Did you see Nick's new haircut? It looks awful."<br>*Nick* (enters): "Mary, how do you like my haircut?"<br>*Mary*: "It looks terrific on you!" |

### 3.2. *FTD*

Patients with FTD performed more poorly than their healthy control group on comprehension of sarcastic remarks only on the 'minimal' subtest ($U = 8.5$, $p < 0.001$) and on lies ($U = 5.5$, $p < 0.005$); there was no group difference on sincere ($U = 27.0$, $p > 0.05$) and paradoxical ($U = 27.0$, $p > 0.05$) conditions, nor on the enriched scenarios of sarcasm ($U = 25.5$, $p > 0.05$).

## 4. Discussion

We examined social cognition in two groups of patients with known frontal and temporal dysfunction in an attempt to elucidate the cognitive mechanisms involved in the difficulties these patient groups have with social interactions. More specifically, we expected that any differences in the pattern of deficits between these two groups might help to elucidate the point in the process of ToM in which there is a breakdown.

We found both similarities and differences in the response patterns of patients with schizophrenia and those with FTD. Specifically, neither group demonstrated deficits in the perception of sincere or literal interactions; thus, we ruled out the possibility that any difficulties on ToM subtests could be attributed to a generalized problem with comprehension. Interestingly, performance patterns diverged on the ToM tasks. While patients with schizophrenia performed poorly on the perception of sarcastic and paradoxical remarks, as well as of lies, regardless of whether or not the statements were enriched with additional information, we found circumscribed deficits in patients with FTD. The latter groups' difficulties were limited to the perception of sarcasm when only paralinguistic cues (prosody and/or the facial expressions) of sarcasm were available. Their difficulty perceiving lies may reflect problems reconciling contradictory yet apparently literal information, leading them to interpret the most recent information (i.e., the actual lie) literally. In contrast, these patients were able to perceive the intention of the speaker on the sarcasm-enriched items, perhaps because they were able to rely on additional verbal information indicating the speaker's true beliefs and intentions. A similar pattern emerged on the paradoxical items.

One potential limitation to the study design, namely, the possibility of an order effect on the present results, warrants mention. Ideally, we would have presented the conditions in counterbalanced order across participants in each group. Despite the fact that we did not do this, however, we believe that we can safely rule out an order effect. This is certainly the case for the schizophrenia group, as they performed better on the first (sincere or literal) condition than on any of the subsequent conditions. In a similar fashion, the FTD group performed better on the first and third (paradoxical) conditions than on either of the sarcastic conditions (presented second and fourth in order) or the lies (last) condition.

Based on the aforementioned findings, we suggest that the differential pattern of performance of the FTD group on the conditions of the ToM tasks was due to the amount and type of information available. When given additional verbal information, such as a prologue, or paradoxical information, patients with FTD were able to benefit from this and respond correctly. Without this information, they were unable to use paralinguistic cues effectively.

The differential pattern of performance of our two patient groups highlights the broad range of difficulties with interpersonal interactions in schizophrenia and a partially preserved ability in FTD. These discrepant patterns may reflect group differences in illness duration, as well as in level of severity of their respective disorders. Clearly, in light of the relatively young age of onset of schizophrenia, our schizophrenia group had a much longer duration of illness; in fact, some studies have suggested less than normal interpersonal functioning even before symptom onset in schizophrenia [8]. Another interpretation involves the fundamental nature of the two disorders. The neurodevelopmental nature of schizophrenia may impede the process of mastering subtle and effective social skills such as ToM. In contrast, the late onset of FTD, a neurodegenerative disorder, may have allowed these patients to apply previously overlearned skills in perceiving subtle cues in a social interaction, despite their present diagnosis. Consequently, while impaired interpersonal functioning in patients with FTD may reflect a decrement in previously acquired skills, similar deficits in patients with schizophrenia may reflect an altogether inadequately learned process.

Further investigations of social cognition in patients with schizophrenia and those with FTD may help elucidate the cognitive mechanisms involved in these disorders and the reasons for the observed difficulties in social functioning and interpersonal relationships. Shedding light on the pathoaetiology of patients' social difficulties, as well as on the cognitive mechanisms of ToM, may help guide psychotherapeutic efforts to improve social functioning, and, consequently, their quality of life.

## Acknowledgements

This research was supported by a Pythagoras II Grant for the Reinforcement of Research Teams in Universities (Ministry of Education and Religion of the Hellenic Republic) to the first author.
